# Serum *trans*-fatty acids level are positively associated with lower food security among american adults

**DOI:** 10.1038/s41387-017-0008-7

**Published:** 2018-03-07

**Authors:** Mohsen Mazidi, Hassan Vatanparast

**Affiliations:** 1 Key State Laboratory of Molecular Developmental Biology, Institute of Genetics and Developmental Biology, Chinese Academy of Sciences, Beijing, 100101 China; 2 Institute of Genetics and Developmental Biology, International College, The University of Chinese Academy of Science, Beijing, 100101 China; 3 College of Pharmacy and Nutrition/School of Public Health, University of Saskatchewan, Saskatchewan, Canada

## Abstract

**Objectives:**

In the current study we aimed to assess whether the food security is associated with serum *trans*-fatty acids (TFAs) and dietary fat.

**Methods:**

Analyses were restricted to participants (from the US National Health and Nutrition Examination Survey) with data available on serum and diet TFAs and food security status from 2009 to 2010. All statistical analyses (analysis of covariance and linear regression) accounted for the survey design and sample weights.

**Results:**

We included 3876 participants, overall (48.6%) participants were men, and (51.4%) were women, generally (69.0%) had high food security. Subjects with higher food security had a higher level of education as well (*p* < 0.001). Age-adjusted, sex-adjusted, race-adjusted, education-adjusted mean of *trans* 9-octadecenoic acid and *trans*-9, *trans*-12-octadecadienoic acid were higher in plasma of participants with lower food security (all *p* < 0.001), moreover in same model there was a significant positive association between plasma level of *trans*-11-octadecenoic acid, *trans-*9-octadecenoic acid and *trans*-9, trans-12-octadecadienoic acid and score of food security. Further, age, sex, race, education, and energy intake adjusted mean of dietary fatty acids show that total polyunsaturated fatty acids are higher in subjects with higher food security (*p* = 0.026) while, cholesterol consumption is higher in subjects with lower food security (*p* = 0.039).

**Conclusions:**

Our findings provide more evidence on the association between food insecurity and the higher level of TFAs in serum and different type of fat in the diet.

## Introduction

*Trans*-fatty acids (TFAs) are unsaturated fatty acids with at least one unsaturated, non-conjugated double bond in the *trans* (rather than the typical *cis*) configuration. Because humans do not synthesize TFAs, levels of these fatty acid isomers in serum depend on dietary intake. TFAs occur naturally in fat from ruminant animal meat, milk, and dairy fat and artificially in industrially hardened vegetable oils^1^. Increased dietary intake of TFAs is linked with the incident of cardiovascular disease (CVD), diabetes mellitus and atherosclerosis^2–5^. It has been suggested that plasma TFAs concentrations may reflect body fatty acid composition, quality of dietary fat and the type of fat consumed over a long period^6, 7^. A recent observational study^8^ and a short-term randomized controlled trial^9^ have indicated that TFA intake increases systemic inflammation in generally healthy individuals. In addition, it has been reported that systemic inflammation is counted as an independent risk factor for the risk of future CVD^10^, taking together the information can provide us a novel potential insight into the crucial role of the TFAs which may affect the cardiovascular health in generally healthy subjects.

As defined by Food and Agriculture Organization of the United Nations “Food security is a situation that exists when all people, at all times, have physical, social and economic access to sufficient, safe and nutritious food that meets their dietary needs and food preferences for an active and healthy life”^11^. The data from the 2013 Current US Population Survey showed that, nationally, 15.8% of people were food insecure^12^. Food security exhibits itself in a limited capability to buy healthy foods (e.g., vegetables, fruits, and animal products) because of their greater price compared to fast foods and junk foods high in fat and added sugar (cheaper)^13, 14^. Latin–American studies have found an association between food security and poorer dietary quality among adults, particularly low intake of fruits, vegetables, meat, and dairy products^14–16^. Currently, food security counted as one of the main social determinants of health^17^.

Food insecurity, as a social determinant of health, is another marker of socioeconomic distress that has been associated with CVD risk factors and other outcomes in recent years^18–21^. However, the underlying mechanism is not yet known. As there is a strong association between food consumption patterns and some non-communicable diseases such as metabolic syndrome (MetS)^22–24^ and CVD^25–27^, the inexpensive, low quality of food commonly used in food insecure households could be a link between food security with Mets and CVDs.

Given to importance of the role of TFAs via inflammation in the pathology of CVD and atherosclerosis, we hypothesize that individuals with higher food security should possibly have a lower level of TFAs in their serum reflecting their consumption pattern of fatty acids consumption. Our results could shed light to better understand the pathways that explain the reported association between food security and CVD, atherosclerosis, and MetS^28–30^. A better understanding of the association between food security and serum TFAs can potentially inform policy makers and dietitians to direct interventions facilitate availability and accessibility of high-quality food fro at risk population. Therefore, we aimed to examine the association between food security status with serum TFAs and also fatty acids intake by using a nationally representative sample of American adults.

## Materials and methods

This is a cross-sectional study using the publically available National Health and Nutrition Examination Survey (NHANES) data set. NHANES is an ongoing survey of a nationally representative sample of the US population with details available elsewhere^31–35^. All participants were requested to provide informed consent, and the National Centre for Health Statistics Research Ethics Review Board approved the protocol. Since the data is publically available, the study was exempted from obtaining additional local institutional ethics review board approval. We combined four 2-year NHANES survey cycles from 2009 to 2010. We Included subject with higher or equal to 18 years of age. Details on NHANES Laboratory/Medical technologists procedures, anthropometry, laboratory procedures, collection, storage, calibration and quality control of blood samples are described elsewhere^35–37^. (http://www.cdc.gov/NCHS/data/nhanes/nhanes_09_10/CRP_F_met.pdf. [accessed 19.08.13]).

### Plasma TFAs

A blood sample was drawn from the participant’s antecubital vein by a trained phlebotomist according to a standardized protocol^33, 38^. Plasma TFAs were measured as part of the NHANES protocol and assessed the total (free and esterified) content of selected *TFA*s in plasma and provides results in concentration units^39, 40^. In this method, the fatty acids in plasma are converted into free fatty acids by subsequent acidic and alkaline hydrolysis. Free fatty acids are extracted using liquid-liquid extraction and derivatized with pentafluorobenzyl bromide^39, 41^. The derivatized fatty acids are separated by capillary gas chromatography and detected by mass spectrometry using negative chemical ionization. The fatty acids are identified based on their chromatographic retention time and on the specific mass to charge ratio of the ion formed in the ion source. Retention times are then compared against those obtained with known standards^42^. Quantitation is performed with a standard solution using stable isotope-labeled fatty acids as internal standards. To calculate *TFAs* as a percent of total fatty acids, 29 fatty acids were determined by this measurement procedure. These fatty acids comprise 95% of all fatty acids present in plasma^43^. This method determines the following four *TFAs*: *trans*-9-hexadecenoic acid (palmitelaidic acid, C16:1n-7t), *trans*-9-octadecenoic acid (elaidic acid, C18:1n-9t), *trans*-11-octadecenoic acid (vaccenic acid, C18:1n-7t-), *trans*-9, *trans*-12-octadecadienoic acid (linolelaidic acid, C18:2n-6t, 9t)^39^. More detailed information on the NHANES protocol can be found in NHANES manual. (https://www.cdc.gov/Nchs/Data/Nhanes/Nhanes_99_00/TFA_A_trans_fatty_acids_met.pdf.)

### Food security

More than 99% of the eligible sample participated in the Food Security Survey Module, which is a well-validated questionnaire developed by the US Department of Agriculture (USDA) to measure household food security over the prior 12 months^44^. Using validated cut points, we categorized the subjects as (a) adult full food security: no affirmative response in any of these items, (b) Adult marginal food security: 12 affirmative responses. (c) Adult low food security: 3–5 affirmative responses, (d) Adult very low food security: 6–10 affirmative responses^44^.

### Statistical analysis

We used the guidelines set by the Centers for Disease Control and Prevention for analysis of the NHANES data set, to analyze the data accounting for the masked variance and using their suggested weighting methodology^35, 45^. Age-, education-adjusted, sex-adjusted and the race-adjusted mean of plasma TFAs were compared across categories of food security (high food security vs. food insecurity) using analysis of covariance (ANCOVA). Adjusted (age, sex, education, and race) linear regression applied to examine the association between food security score with plasma TFAs levels. All tests were two-sided, and alpha was set at *p* < 0.05 presenting the level of significance unless otherwise stated. We have applied complex sample strategies, to deal with sample weights, unequal probabilities of selection, nonresponse bias, and oversampling. SPSS software (version 18, Chicago, IL, USA) was used for statistical analysis.

## Results

A total of 10,537 participants took part in NHANES across the years under consideration, of which 3876 were eligible for inclusion in the current analyses. From the eligible sample, (69.0%) had high food security. Overall (48.6%) participants were men, and (51.4%) were women. Compared to those with high food security, participants with low food security comprised more of the minority ethnic groups including Mexican-Americans (27.4 vs. 14.9%), other Hispanic (14.3 vs. 8.2%), non-Hispanic Black (23.0 vs. 16.2%), and fewer non-Hispanic White (30.4 vs. 54.7%); *p* < 0.001 for differences in the distribution of ethnicity by status for food security. The mean age was 48.1 years overall; and the age was higher in participants with high food security than in those without (50.5 vs. 42.7 years, *p* < 0.001). Participants with high food security were mostly in group with higher education level, “less than high school” (high food security = 8.6% vs. low food security = 20.9%), “completed high school” (high food security = 35.3% vs. low food security = 47.7%) and “more than high school” (high food security = 55.9% vs. low food security = 31.2%); *p* < 0.001 for differences in the distribution of education level by status for food security.

Using ANCOVA, we computed adjusted (age, sex, race, education) mean of the plasma TFAs for the level of the food security. The level of *trans*-9-octadecenoic acid and *trans*-9, *trans*-12-octadecadienoic acid was higher in plasma of participants with lower food security (all *p* < 0.001, Table 1).

**Table 1:** Adjusted mean of plasma *trans* fatty acids and risk of food insecurity

Variables		Food security status		*P*-value	*Β* coefficient	*P*-value
		High	Margin + low + very low			
Plasma	*Trans* 9-hexadecenoic acid (umol/l)	4.02 ± 0.11	4.14 ± 0.10	0.459	0.004	0.851
	*Trans* 11-octadecenoic acid (umol/l)	19.2 ± 0.50	20.3 ± 0.82	0.214	0.044	<0.001
	*Trans* 9-octadecenoic acid (umol/l)	15.8 ± 1.03	17.4 ± 1.20	<0.001	0.061	<0.001
	*Trans*-9, *trans*-12-octadecadienoic acid	1.70 ± 0.03	2.12 ± 0.03	<0.001	0.056	<0.001
Diet	Total fat (g)	72 ± 1.9	70 ± 3.1	0.762	−0.140	0.283
	Total saturated fatty acids (g)	22 ± 0.66	23 ± 1.3	0.415	0.003	0.810
	Total monounsaturated fatty acids (g)	26.2 ± 0.7	26.6 ± 1.2	0.834	−0.014	0.271
	Total polyunsaturated fatty acids (g)	16.2 ± 0.6	15.1 ± 1.1	0.026	−0.035	<0.001
	Cholesterol (mg)	290 ± 17.2	299 ± 20.3	0.039	0.011	0.380

Analysis of covariance applied to calculate the adjusted (age, sex, race, education) mean. Linear regression adjusted for (age, sex, race, education). Value expressed as a mean and SEM

There was a significant positive association between plasma level of *trans*-11-octadecenoic acid, *trans-*9-octadecenoic acid and *trans*-9, *trans*-12-octadecadienoic acid and score of food security, that is, subjects with a higher score, lower food security, have a higher level of TFAs in plasma. Further, age, sex, race, education, and energy intake adjusted mean of dietary fatty acids show that total polyunsaturated fatty acids (PUFA) are higher in subjects with higher food security (*p* = 0.026, Table 1) while cholesterol consumption is higher in subjects with lower food security (*p* = 0.039, Table 1). There was also a significant association between dietary PUFA and the level of food security (*β* = −0.035, *p* < 0.001, Table 1).

## Discussion

The present study evaluates the association between plasma TFAs level with food security in US population. Significant and inverse association between plasma level of *trans*-11-octadecenoic acid, *trans-*9-octadecenoic acid, and *trans*-9, *trans*-12-octadecadienoic acid and food security is the main finding of this study. In addition, the consumption of PUFA was higher in participants with higher food security status.

In line with our findings, an investigation in US population reported that food-insecure adults have a higher consumption of high-dairy products. Also, food insecurity was significantly associated with lower Healthy Eating Index, consequently leading to increasing chronic disease risk^46^. In another cross-sectional study from the California Health Interview Survey, food insecurity was significantly associated with lower consumption of fruits and vegetable^47^. In addition, food-insecure individuals had a high total fat intake^48^.

The tool used in NHANES, Household Food Security Survey Module, is measuring income-related household food security. This reflects the fact that disadvantaged groups of the population who have lower socio–economic status are more at risk of food insecurity. A study among Vietnamese reported that food insecurity was inversely associated with total household income and fruit intakes^49^. The cost of high-quality food such as fresh fruit and vegetable is the main factor limiting in at-risk population leading to the consumption of more fast food and junk foods high in *trans*-fat and saturated fat and sugar and low in micronutrients intake leading to a higher prevalence of inadequacy in nutrient intake and iron deficiency anemia^50^. Conversely, foods with high calorie which mostly consumed by those with low food security might contribute to obesity, and raise the likelihood of the NCDs, such as T2D and CVD^50^. Recent reports from NHANES 2003–2010 stated that food-insecure adolescents consume more fast foods in comparison with the food-secure population^51^. It has been reported that the high intake of the foods that are loaded with a great amount of the saturated fats may lead to deterioration of the blood lipid profile. Moreover, in study conducted in adolescents, a higher odds of the having elevated Total cholesterol was reported in participants with diets that was highly loaded with sugars including sucrose and fructose^52^.

While the association between food insecurity and chronic conditions has been reported in epidemiologic studies^18–20^, the pathophysiologic pathway is not clear. Our finding that food insecure adults have a higher serum level of TFAs compared to food secure individuals may partly explain such association due to the pro-inflammatory role of TFAs. Therefore, public awareness, initiatives, and policies that limit the TFAs level in diet can be a reasonable approach for improving diet quality, in general, and among food insecure households in particular, and preventing its related diseases.
